# Misdiagnosis of chronic kidney disease and parathyroid hormone testing during the past 16 years

**DOI:** 10.1038/s41598-023-43016-x

**Published:** 2023-09-22

**Authors:** Haojie Liu, Huan Zhao, Danna Zheng, Wenfang He, Yueming Liu, Juan Jin, Qiang He, Bo Lin

**Affiliations:** 1 The 2Nd Clinical Medical College, Zhejiang Chinese Medical University, Hangzhou, China; 2 Urology and Nephrology Center, Department of Nephrology, Zhejiang Provincial People’s Hospital (Affiliated People’s Hospital, Hangzhou Medical College), Hangzhou, China; 3 Department of Nephrology, the First Affiliated Hospital of Zhejiang Chinese Medical University (Zhejiang Provincial Hospital of Traditional Chinese Medicine), Hangzhou, 310000 Zhejiang China; 4 Department of Nephrology, Zhejiang Provincial People’s Hospital Bijie Hospital, Guizhou, 551700 China

**Keywords:** Endocrinology, Nephrology

## Abstract

Chronic kidney disease (CKD) is a prevalent pathological condition worldwide. Parathyroid hormone (PTH) is an important index related to bone metabolism in CKD patients and has not received enough attention. This study was performed to investigate the incidence and diagnostic rate of CKDin hospital as well as PTH testing and treatment for secondary hyperparathyroidism (SHPT) in patients with stage 3 to 5 CKD. The data of patients who visited Zhejiang Provincial People's Hospital from February 2006 to April 2022 were retrieved from the hospital database. All data were divided into three subgroups using PTH testing and SHPT treatment as major comparative indicators for analysis. The data were then analyzed for overall PTH testing, CKD incidence, and diagnostic rate. Among 5,301,391 patients, the incidence of CKD was 13.14%. The missed diagnosis rate for CKD was 65.76%. The total PTH testing rate was 1.22%, of which 15.37% of PTH testing was performed in patients with stage 3 to 5 CKD. The overall diagnosis rate of SHPT in patients with stage 3 to 5 CKD was 31.0%. The prophylactic medication rate was 7.4%, and the rate of post-diagnostic drug therapy was 22.2% in patients who underwent SHPT treatment. The high misdiagnosis rate and low PTH testing rate of CKD requires prompt attention from clinicians. SHPT treatment should be considered especially in patients with stage 3 to 5 CKD.

## Introduction

The incidence of chronic kidney disease (CKD) has been increasing worldwide and is regarded as a public health concern requiring greater attention^1^. The global incidence of stage 3 to 5 CKD was 10.6% in 2016^2^. Moreover, a recent cross-sectional study in China showed the prevalence of CKD in Chinese adults was 8.2%^3^. Although CKD patients who have visited the hospital undergo extensive examinations for kidney disease, the diagnosis of CKD is still missed in many patients.

Parathyroid hormone (PTH) is a peptide-derived hormone secreted from the chief cells in the parathyroid gland. It is a major modulator of bone and mineral metabolism and a key regulator of calcium homeostasis^4^. PTH plays a vital role in regulating the balance of calcium and phosphorus metabolism by promoting calcium reabsorption in the renal tubules, calcium absorption in the gastrointestinal tract, bone calcium mobilization, and phosphate excretion in the kidney^5^. The synthesis and secretion of PTH are mainly regulated by extracellular calcium, which binds and activates calcium-sensitive receptors on parathyroid cells, thereby reducing PTH release^6^.

Secondary hyperparathyroidism (SHPT) is a type of hypermetabolism caused by underlying primary diseases, such as chronic renal failure and post-hemodialysis. The low blood calcium caused by these diseases induces over-secretion of PTH^7^. SHPT is a consequence and critical mediator of CKD–mineral bone disorder (CKD-MBD)^8^. The pathogenesis of SHPT is complex and driven by many factors, including deficiency of 1,25-dihydroxyvitamin D, hypocalcemia, and hyperphosphatemia^9,10^; CKD is regarded as the primary etiology. The imbalance in the fibroblast growth factor (FGF) –calcium–phosphate–vitamin D–PTH axis translates into vascular calcification and bone dystrophy in patients with stage 3 CKD, resulting in a low estimated glomerular filtration rate (eGFR)^11,12^. However, recent studies have shown that low PTH levels (less than twice the upper limit of normal) in patients with end-stage kidney disease are also associated with higher mortality risk^13,14^.

The exploration of CKD-MBD, which is associated with hyperphosphatemia, hypocalcemia, low serum levels of vitamin D, and increased PTH secretion, has gradually increased in recent years^15^. SHPT is one of the most important clinical manifestations of CKD-MBD, and PTH is also a crucial indicator of prognosis in CKD^8,11,12^. However, there are some indications that clinicians do not seem to pay as much attention to the diagnosis of CKD and the detection of PTH. We aim to amplify this phenomenon and discuss it by analyzing a large sample of data.

The present study was performed to investigate the incidence and diagnostic rate of CKD, the rate of PTH testing, and the status of SHPT treatment in patients with stage 3 to 5 CKD in a single hospital in China over 16 years.

## Methods

### Patients and data collection

**Figure 1:**
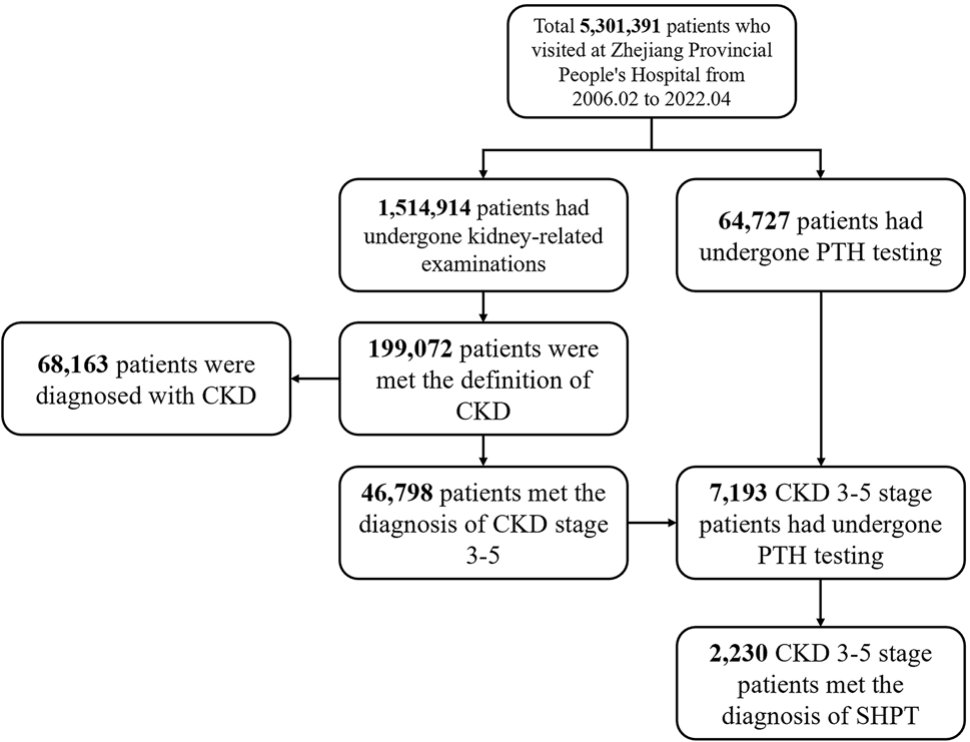
Flow of participants.

The data of all patients who visited Zhejiang Provincial People's Hospital from February 2006 to April 2022 with recorded general and medical data were retrospectively retrieved from the hospital records, and the data were scrutinized for the patients’ general information, laboratory data, and drug treatment. The inclusion criteria were abnormal renal function lasting > 3 months, abnormal kidney imaging examination findings lasting > 3 months and an eGFR of ≤ 60 for > 3 months. Patients who had undergone kidney transplantation were also eligible for inclusion. Patients who had been diagnosed with acute renal insufficiency were excluded from the study. 199,072 patients were selected and included in the CKD group. The study flow chart is shown in Fig. 1.

This study was conducted according to the Declaration of Helsinki principles. This is a retrospective analysis using Zhejiang Provincial People’s Hospital record database. The study protocol was approved by the Institution Ethics Committees of Zhejiang Provincial People’s Hospital and granted a waiver of written informed consent.

### Definitions and explanations

The eGFR was calculated using the CKD Epidemiology Collaboration formula, and the diagnosis of stage 3 to 5 CKD was made using the 2021 Kidney Disease: Improving Global Outcomes (KDIGO) guidelines as a standard (eGFR of < 60 mL/min/1.73 m^2^)^16^. The KDIGO 2021 guidelines were further used for the diagnosis of CKD^16^. Similarly, the diagnosis of hyperparathyroidism was based on the 2021 KDIGO guidelines: PTH of > 70 pg/mL was the diagnostic criterion for stage 3 CKD, PTH of > 110 pg/mL for stage 4, and PTH of > 300 pg/mL for stage 5^16^.

### Research methods

The data were analyzed for the overall incidence of CKD and the rate of PTH testing in the total cohort, and patients who had undergone kidney-related examinations were screened. Eligible patients who met the inclusion criteria were selected. The incidence and diagnosis rates of CKD were calculated. After analysis of the overall and department-specific PTH testing rates, all selected patients with CKD were divided based on the eGFR, and the data of patients with stage 3 to 5 CKD were obtained for PTH-related studies. And the annual increase in the number of PTH tests and patients with stage 3 to 5 CKD was calculated. All selected patients were divided into three groups: those with stage 3 to 5 CKD (Group A), those with stage 3 to 5 CKD who had undergone PTH testing (Group B), and those with stage 3 to 5 CKD who had been diagnosed with SHPT (Group C). All patients' general and clinical data were collected and analyzed for age, sex, visit type, CKD stage, and the use of drugs for SHPT treatment, including calcium supplements, active vitamin D3, lanthanum, sevelamer and cinacalcet.

### Statistical analysis

All data were statistically analyzed using SPSS version 25 (IBM Corp., Armonk, NY, USA), and figures were drawn using GraphPad Prism version 9 (GraphPad Software, San Diego, CA, USA). Measurement data with a normal distribution are expressed as mean ± standard deviation. Histograms and line graphs were developed for the visual expression of the data.

## Results

### Incidence and diagnosis of CKD

**Figure 2:**
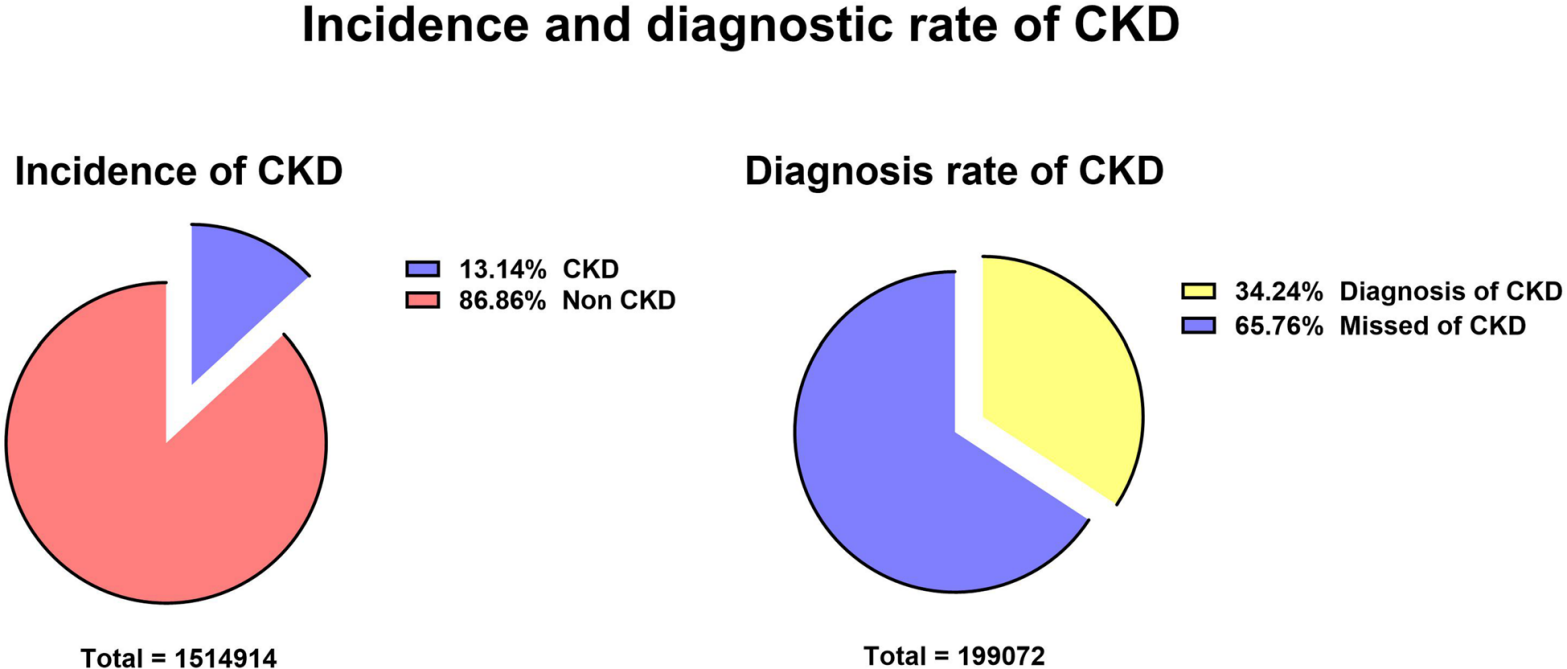
Incidence and diagnostic rate of CKD.

During the selected study period, 5,301,391 patients visited Zhejiang Provincial People’s Hospital, among whom 1,514,914 were examined for kidney-related disorders. Among these patients, 199,072 satisfied the definition of CKD. The incidence of CKD was 13.14%, among whom 68,163 patients were diagnosed with CKD (diagnostic rate of 34.24%). The data regarding the incidence and diagnosis of CKD are shown in Fig. 2.

### Overall and department-specific PTH testing rates

**Figure 3:**
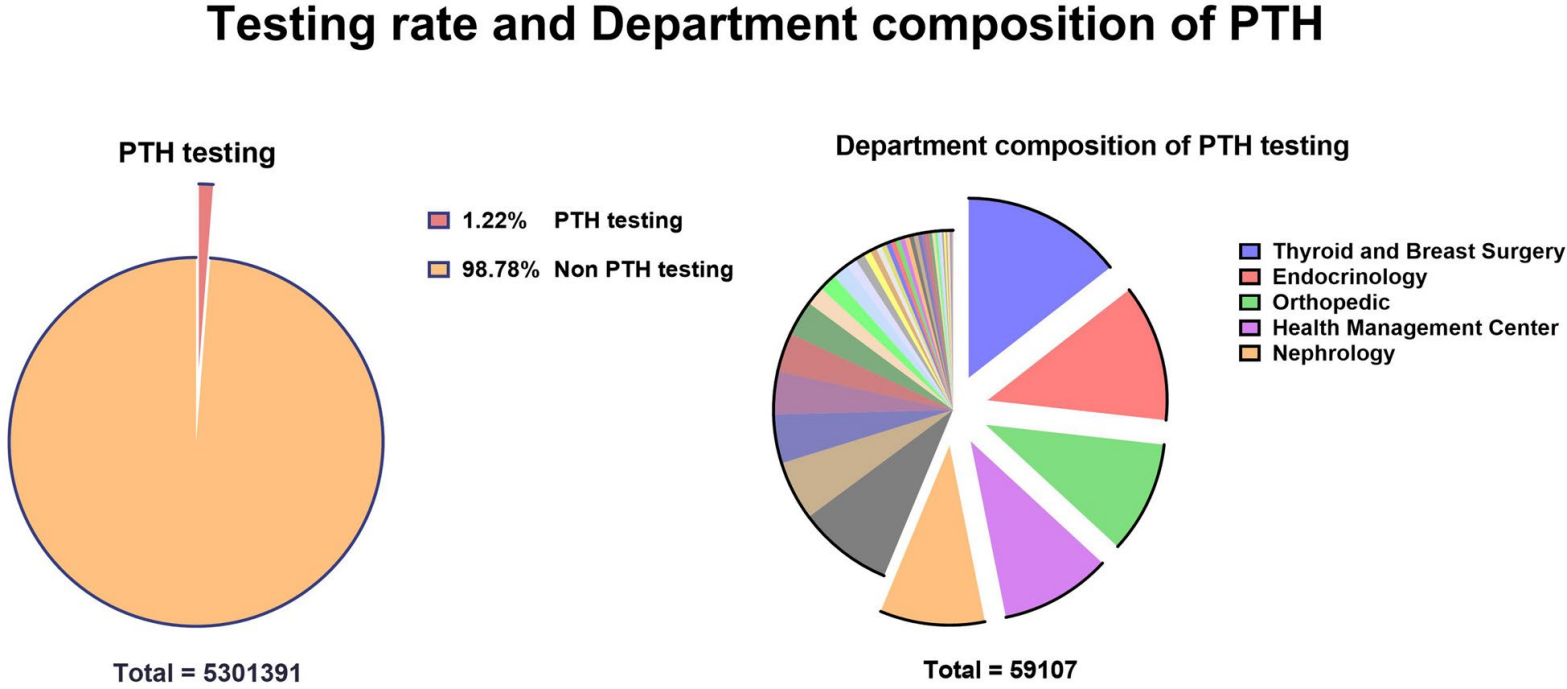
Testing rate and Department composition of PTH.

Among all selected patients, 64,727 patients underwent PTH testing (testing rate of only 1.22%). The PTH testing situation at the departmental level was then analyzed. The number of PTH tests from each department during the study period was obtained, resulting in 59,107 patient records in the database. Among the screened departments, the top five were Thyroid and Breast Surgery, Endocrinology, Orthopedics, Health Management Center, and Nephrology. Head and Neck Surgery, which ranked sixth, was not far behind the top five. All the other departments had a far lower number of PTH tests than the top six. These results are shown in Fig. 3.

### General information of patients in the three study groups

As shown in Table 1, Group A had a significantly higher proportion of men (59.0%) than women (41.0%), whereas no significant difference in sex was observed in Group B or C. The mean age of the patients in Groups A, B, and C was 74.2 ± 15.7, 69.7 ± 17.2, and 66.2 ± 18.0 years, respectively. Regarding the types of visits (outpatient vs. inpatient), only the first PTH test was counted in Group B, which resulted in the detection of 6,652 (92.5%) patients during hospitalization. In comparison, only 541 (7.5%) patients were tested in the outpatient and emergency departments.

**Table 1:** Basic information of patients in groups A, B, and C.

		A (n = 46,798)	B (n = 7193)	C (n = 2230)
Gender, No (%)	Male	27,616 (59.0)	4242 (59.0)	1306 (58.6)
	Female	19,182 (41.0)	2951 (41.0)	924 (41.4)
Age, mean (SD), y	–	74.2 (15.7)	69.7 (17.2)	66.2 (18.0)
Visit type, No (%)	Outpatient	–	541 (7.5)	–
	Inpatient	–	6652 (92.5)	–
CKD stage, No (%)	G3	29,321 (62.7)	2961 (41.2)	694 (31.1)
	G4	8922 (19.1)	1148 (16.0)	428 (19.2)
	G5	8555 (18.3)	3084 (42.9)	1108 (49.7)
Medication used, No (%)	Before diagnosis	–	–	164 (7.4)
	After diagnosis	–	–	496 (22.2)

In Group A, stage 3, 4, and 5 CKD was present in 29,321 (62.7%), 8922 (19.1%), and 8555 (18.3%) patients, respectively. In Group B, stage 3, 4, and 5 CKD was present in 2961 (41.2%), 1148 (16.0%), and 3084 (42.9%) patients, respectively. In Group C, stage 3, 4, and 5 CKD was present in 694 (31.1%), 428 (19.2%), and 1108 (49.7%) patients, respectively. The proportion of patients with stage 5 CKD was significantly higher among patients who had undergone PTH testing.

### PTH testing in patients with stage 3 to 5 CKD

**Figure 4:**
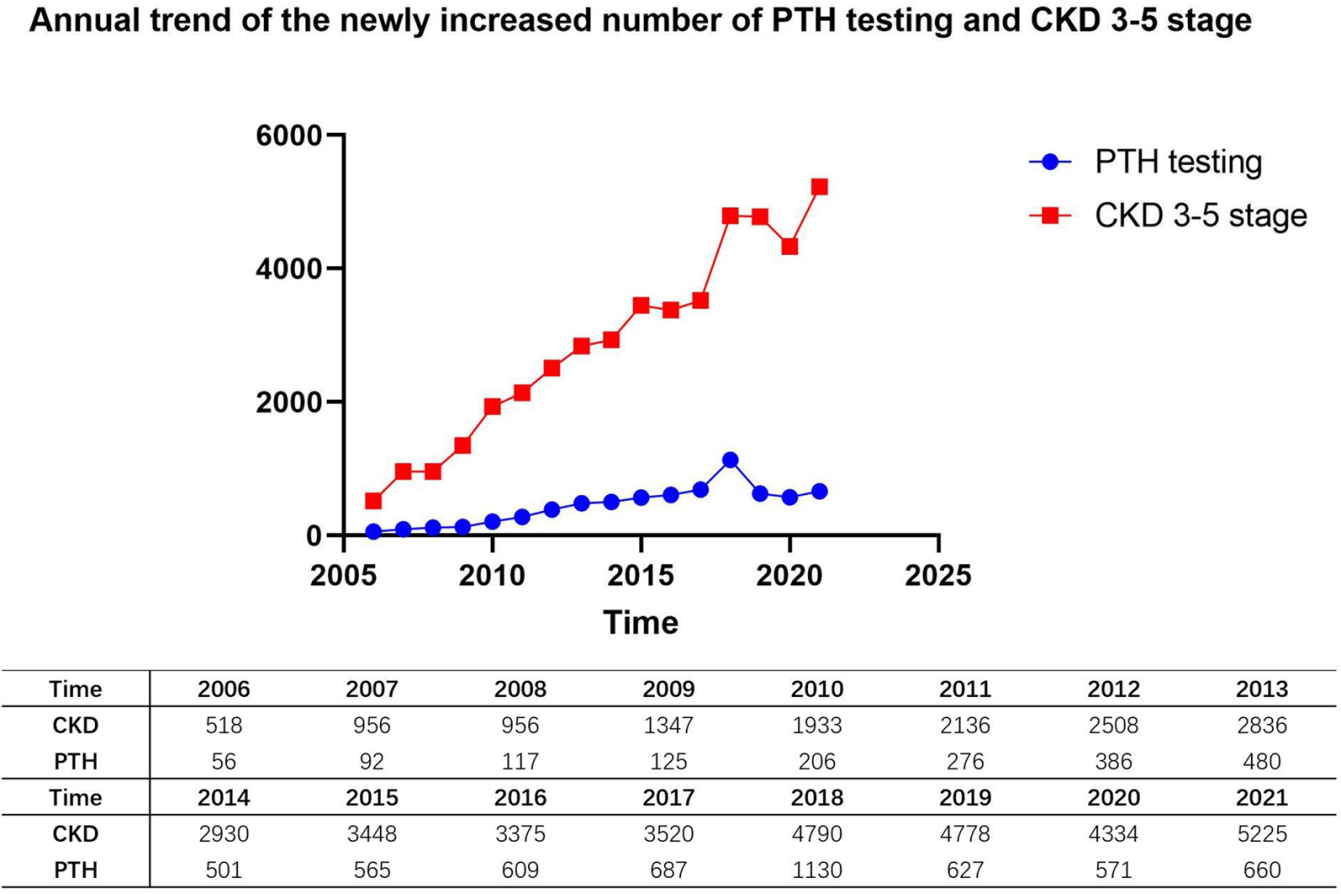
Annual trend of the newly increased number of PTH testing and CKD 3–5 stage.

Combined with the primary data, the annual rate of new PTH testing in patients with stage 3 to 5 CKD was calculated and plotted as a function of time (Fig. 4). Overall, the curve showed an upward trend, and the overall testing rate of patients with stage 3 to 5 CKD was 15.4%. Furthermore, the independent analysis of each stage of CKD demonstrated a 10.1% testing rate for stage 3, 12.9% for stage 4, and 36.0% for stage 5.

### Medications used and diagnosis of SHPT

The drugs used and diagnosis of SHPT were analyzed, and the data are presented in Table 1. We used the time of the first diagnosis of SHPT to divide the drug treatments into two groups: prophylactic and post-diagnostic. The medications used before the diagnosis of SHPT were considered prophylactic, with a prophylaxis rate of 7.4%, while the post-diagnosis drug treatment rate was 22.2%. We made detail statistics on the medications used of prophylactic and post-diagnosis in Table 2. As can be seen in Table 2, calcium and active vitamin D were most used drugs, and the use of all medications increased significantly after diagnosis. The diagnosis rate of SHPT in patients with stage 3 to 5 CKD was also calculated, and the results showed that the overall diagnosis rate was 31.0%. The diagnostic rate of SHPT in patients with stage 3, 4, and 5 CKD was 23.4%, 37.3%, and 36.0%, respectively.

**Table 2:** Prophylactic and post-diagnostic medication used.

	Prophylactic	Post-diagnostic
Calcium, No (%)	104 (4.67)	331 (14.84)
Lanthanum, No (%)	9 (0.40)	89 (3.99)
Active vitamin D, No (%)	114 (5.11)	399 (17.89)
Sevelamer, No (%)	31 (1.39)	149 (6.68)
Cinacalcet, No (%)	6 (0.27)	30 (1.35)
Medication used*, No (%)	164 (7.35)	496 (22.24)

*Some patients use multiple drugs at the same time.

## Discussion

In this study, the incidence and diagnostic rate of CKD were investigated in patients who visited Zhejiang Provincial People's Hospital from February 2006 to April 2022 by retrospectively retrieving the patients’ data from the hospital database. The data showed that the incidence of CKD was significantly higher than the data reported in 2016 and 2023^2,3^.

The patients who visited the hospital had poorer health conditions with a higher risk of CKD than those in the regional cohort. Surprisingly, the proportion of patients with a missed diagnosis of CKD exceeded 65%. A possible reason for this high rate is a lack of familiarity with CKD diagnosis in other departments^17^. Another possible reason is the acute reversible kidney injuries (e.g., recurrent kidney stones) as the cause of the examination abnormalities.

The focus of this study differs from that of previous cross-sectional studies in different provinces and cities in China^18,19^ in the respect that we investigated and analyzed the overall testing rate of PTH. Our results showed that this rate was only 1.22% during the study period (Fig. 2), indicating that PTH has been neglected as an indicator of CKD. Our data showed that the Thyroid and Breast Surgery Department was the most common department conducting PTH testing during the past 16 years. PTH is a hormone with systemic action; hence, patients with hormonal disturbances usually prefer to visit the Endocrinology Department as their first choice. Furthermore, PTH testing is also performed during orthopedic examinations because PTH is closely associated with osteoblast and osteoclast activities, which determine growth and bone development^20^. The PTH testing rate in the Health Management Center unexpectedly ranked fourth, reflecting the importance of PTH measurement as a physical examination tool. In the Nephrology Department, PTH is often measured in patients with CKD because these patients often have CKD-MBD^21^. China has a well-developed health insurance system. PTH testing as a basic treatment item is allowed to be fully reimbursed. There is no financial burden on the patients. Thus, we don’t think this is one of the reasons for the low PTH testing.

This study involved patients with stage 3 to 5 CKD. A Japanese study showed that a reduction in the eGFR to < 60 mL/min/1.73 m^2^ with deficient vitamin D is a significant risk factor for an increased serum PTH concentration^22^. To highlight a more typical effect, we selected more representative sample data.

We found no significant sex difference among patients with stage 3 to 5 CKD who had SHPT. Concerning age, the patients with stage 3 to 5 CKD who underwent PTH testing were relatively younger than those who did not undergo testing, probably because younger patients paid more attention to PTH testing. Analysis of the types of visits among patients first tested for PTH showed that many patients were tested during hospitalization. PTH testing takes a long time to perform. If the test results cannot be made available to the attending physician on the same day of the outpatient visit, the complexity of outpatient treatment is significantly increased. Furthermore, outpatient testing programs are not comprehensive, and only the necessary tests are often selected for the primary assessment of the condition. During hospitalization, however, PTH is used for a comprehensive assessment of the patient’s condition.

Our data showed that the overall PTH testing rate in patients with stage 3 to 5 CKD was only 15.4%. Patients with stage 3 to 5 CKD develop severe parathyroid gland function insufficiency, resulting in loss of the metabolic regulation ability of calcium, phosphorus, and active vitamin D3. Moreover, numerous clinical trials have confirmed that high PTH is a critical risk factor for the prognosis of patients with CKD, directly leading to an increased risk of cardiovascular disease^23,24^. The testing rate of 15.4% indicates that the parathyroid function of patients with CKD demands more attention.

In the present study, the PTH testing rates of patients at different stages of CKD were grouped, and the results demonstrated that patients with stage 3, 4, and 5 CKD had a testing rate of 10.1%, 12.9%, and 36.0%, respectively. These low PTH testing rates for patients with stage 3 and 4 CKD indicate that PTH testing in patients with early CKD still requires more attention. A retrospective study in the United States showed that only 46% of patients with stage 4 CKD and 41% of those with stage 5 underwent PTH testing^25^. These data suggest that the PTH testing rates in patients with CKD worldwide are concerning. The statistical analysis of our data for patients with stage 3 to 5 CKD from February 2006 to April 2022 showed that the annual trend of newly detected patients generally increased yearly (Fig. 4). This shows that as in-depth research on CKD increased, clinicians began to pay more attention to PTH.

Further statistical analysis revealed that the diagnosis rate for SHPT in patients with stage 3 to 5 CKD was 31.0%. The prevalence of SHPT in CKD has been well described, with estimates ranging from 20 to 80% depending on the severity of CKD^26^. A recent study conducted in Italy showed a 38% incidence of SHPT in patients with stage 3 to 5 CKD^27^. By contrast, in our study, the diagnosis rate of SHPT was 23.4%, 37.3%, and 36.0% in patients with stage 3, 4, and 5 CKD, respectively (Table 1). The meager testing rate of PTH indicates that many patients with CKD who possibly have SHPT have yet to be detected and diagnosed promptly, which significantly impacts the prognosis of patients with CKD.

Based on the pathophysiology of SHPT, a variety of preventive and therapeutic approaches can be used, including reducing phosphorus intake, using phosphate binders, and using active vitamin D or calcimimetics^28^. In recent years, the use of active vitamin D and cinacalcet has significantly increased and the performance of parathyroidectomy has decreased^29–31^. As shown in Table 1, 7.35% of patients who had stage 3 to 5 CKD with SHPT had received prophylaxis before the diagnosis of SHPT, whereas the proportion of patients who received medications increased to 22.24% following the diagnosis of SHPT. This could be due to a lack of attention to calcium and phosphorus metabolism disorders in patients with CKD.

Furthermore, although the risk of SHPT is higher in patients with stage 3 to 5 CKD, some patients’ serum calcium and phosphorus levels remain within the normal range, discouraging therapy use^32^. The KDIGO guidelines also do not recommend routine use of calcitriol or its analogs in patients with stage 3 to 5 CKD because of an increased risk of hypercalcemia^9^. Moreover, dialysis can partially regulate electrolyte disturbances in the body, which may have some influence on the choice of drug therapy. An additional reason for low drug treatment could be the availability of a wide variety of calcium supplements on the market that is convenient for patients to purchase themselves, resulting in hospitals not prescribing drugs. In detail use of medications of SHTP, the calcium and active vitamin D were used most frequently. The reasons might be cheaper prices and more accessible.

This study had several limitations. First, the data in this study were obtained from a single center. Although the center had a strong regional representation, the data source was still a single institution, and there may have been some sampling errors. Second, some data from the data platform were incomplete, which may have caused some errors. Finally, drug therapy for SHPT only came from the single center involved in the study, and the records of some drugs used outside the hospital were incomplete.

## Conclusion

The clinical situation regarding the diagnosis of CKD, testing of PTH, and treatment of SHPT is not optimistic. The rate of missed diagnosis of CKD is relatively high, requiring clinicians to pay more attention to this disorder. Our data suggest that clinicians should pay more attention to parathyroid function in patients with stage 3 to 5 CKD.

## Data Availability

The datasets used and/or analysed during the current study available from the corresponding author on reasonable request.
